# Context-Dependent Decay of Motor Memories during Skill Acquisition

**DOI:** 10.1016/j.cub.2013.04.079

**Published:** 2013-06-17

**Authors:** James N. Ingram, J. Randall Flanagan, Daniel M. Wolpert

**Affiliations:** 1 Department of Engineering, University of Cambridge, Trumpington Street, Cambridge CB2 1PZ, UK; 2 Department of Psychology and Centre for Neuroscience Studies, Queen’s University, Kingston, ON K7L 3N6, Canada

## Abstract

Current models of motor learning posit that skill acquisition involves both the formation and decay of multiple motor memories that can be engaged in different contexts [1–9]. Memory formation is assumed to be context dependent, so that errors most strongly update motor memories associated with the current context. In contrast, memory decay is assumed to be context independent, so that movement in any context leads to uniform decay across all contexts. We demonstrate that for both object manipulation and force-field adaptation, contrary to previous models, memory decay is highly context dependent. We show that the decay of memory associated with a given context is greatest for movements made in that context, with more distant contexts showing markedly reduced decay. Thus, both memory formation and decay are strongest for the current context. We propose that this apparently paradoxical organization provides a mechanism for optimizing performance. While memory decay tends to reduce force output [10, 11], memory formation can correct for any errors that arise, allowing the motor system to regulate force output so as to both minimize errors and avoid unnecessary energy expenditure. The motor commands for any given context thus result from a balance between memory formation and decay, while memories for other contexts are preserved.

## Highlights

• Motor learning involves the context-dependent formation of motor memories
• In contrast, memory decay has been assumed to be context independent
• We show memory decay is context dependent, being greatest for the current context
• The balance of formation and decay may be optimal for trading off skill and effort

## Results and Discussion

Motor learning has been extensively studied using tasks in which reaching movements are perturbed by applying loads to the arm (for example, [12–16]) or by altering visual feedback of the hand (for example, [17–20]). With practice, subjects adapt to such perturbations, forming motor memories that are expressed as adaptive changes in the motor commands to the arm. This memory formation is context specific. For example, learning in one movement direction or for one object orientation shows limited generalization to other directions or orientations [1, 7, 13, 21–24].

Current models that capture this pattern of motor memory formation [1–9] typically include two update terms that describe how the adaptation state (**z**), which represents motor memories, changes from one trial (n) to the next (n + 1):

(Equation 1) $$z_{n+1}=az_{n}+b_{n}e_{n}.$$

In this case, **z** is a vector of elements that represent the adaptation state associated with different contexts (for example, different movement directions). The scalar retention factor (*a*) determines how much of the adaptation state is carried over from trial to trial. Its value is always less than one, such that the adaptation state tends to decay passively from one trial to the next. The learning rate vector (**b**_n_) determines how strongly the error (*e*_n_) from the current trial is used to update the adaptation states on the next trial. The learning rate vector is context dependent, such that errors have the greatest influence on the state associated with the current context, with diminishing influence on states associated with increasingly distant contexts.

Current models [1–6, 8, 9] thus make two key assumptions. First, error-driven memory formation is context dependent; second, memory decay is context independent. Whereas numerous studies have provided empirical support for context-dependent memory formation [1, 7, 13, 21–24], the assumption that memory decay is context independent has never been directly tested. In the current study, we used a novel experimental approach to examine the effect of context on the decay of motor memories in both an object manipulation and a force-field adaptation task, thus testing the assumption of context independence for the first time.

### Context-Dependent Decay for Familiar Object Dynamics

**Figure 1:**
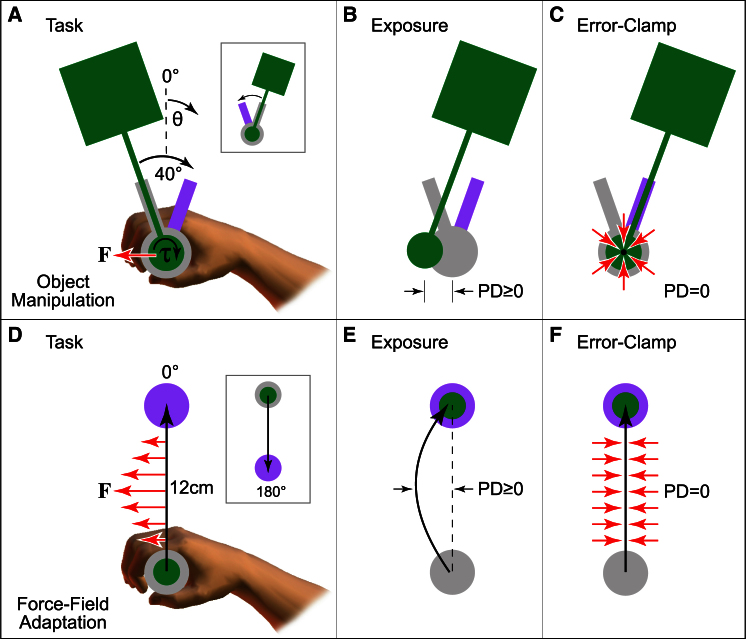
The Object Manipulation and Force-Field Adaptation Tasks (A) Top view of the object manipulation task. Subjects grasped the handle of a robotic manipulandum and rotated a virtual object (green) clockwise and counterclockwise (inset) between visually presented targets (purple), while keeping the handle as still as possible within the central home region (gray). Rotating the object generated forces (**F**, red arrow). (B) On exposure trials, the forces associated with rotating the object caused the handle to displace. The peak displacement (PD) of the handle provided a measure of error on each trial. (C) On error-clamp trials, the manipulandum simulated a 2D stiff spring (red arrows), which prevented displacement of the handle (PD = 0). (D) Top view of the force-field adaptation task. Subjects grasped the handle of a robotic manipulandum and reached with a virtual cursor (green) toward visually presented targets (purple). A velocity-dependent force field (**F**, red arrows) displaced the hand during the movement. Movement to the 0° target is shown (inset shows the 180° target). (E) On exposure trials, the applied forces caused the hand to displace during the movement. The PD of the hand relative to a straight line between the starting position and the target provided a measure of error on each trial. (F) On error-clamp trials, the manipulandum simulated a mechanical channel between the starting position and the target (red arrows), which prevented perpendicular displacement of the hand (PD = 0). See also Figure S1.

In total, 72 university students participated in the study after giving their informed consent. A local ethics committee approved the study, and subjects were naive to its purposes. Subjects grasped the handle of a robotic manipulandum [25] and rotated a virtual object while attempting to keep the grasp point stationary [7, 24] (Figure 1A; see also Figure S1 available online). The task required subjects to produce a compensatory force to oppose the force associated with the circular motion of the mass (**F** in Figure 1A). On a given trial, subjects rotated the object 40° clockwise (CW) or counterclockwise (CCW) between two targets. The performance error was measured as the peak displacement (PD) of the handle (Figure 1B). Adaptation during the task involves forming a motor memory of the magnitude of the compensatory force required to stabilize the handle. The greatest adaptation is observed at the local orientation at which the dynamics are experienced, with limited generalization to novel (untrained) orientations [7, 24]. This pattern of context-dependent memory formation can be reasonably well captured with the standard state-space model (Equation 1) that includes a generalization function tuned to the orientation of the object [7].

**Figure 2:**
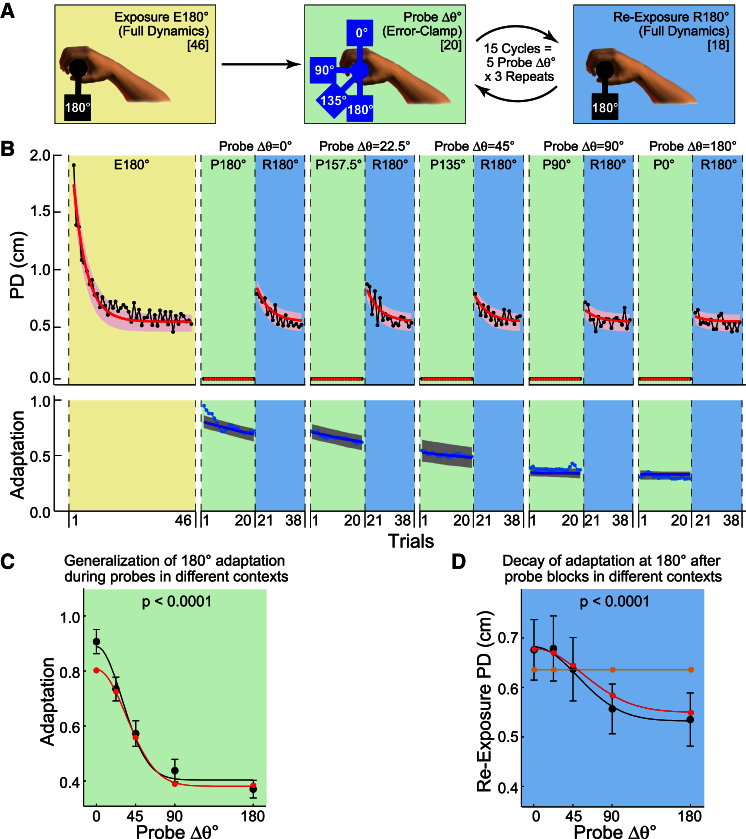
Context-Dependent Decay during Object Manipulation for G180° (A) The experimental paradigm (trial counts in brackets). Subjects were initially exposed to the full object dynamics at the exposure orientation (180°) for 46 trials (E180°, yellow panel). They then performed 15 cycles of alternating probe and reexposure blocks. Probe blocks (green panel) consisted of 20 error-clamp trials presented at one of five probe orientations. Probe orientations included the original exposure orientation (Δθ = 0°) and four nonexposure orientations (Δθ > 0°; probe object at 157.5° is omitted for clarity). Reexposure blocks (blue panel) consisted of 18 trials with full object dynamics at the original exposure orientation (R180°). (B) Composite trial series for peak displacement (PD; upper panels, black trace) and adaptation (lower panels, blue trace) for G180°, including the context-dependent decay model fit (red and dark blue lines; pink and gray shading shows 95% confidence limits for model fit). E, exposure (yellow panel); P, probe (green panels); R, reexposure (blue panels). Δθ° is probe orientation relative to exposure. (C) Generalization of adaptation measured during probe blocks (green panels in A and B) after exposure at 180° (black symbols are mean and SE across subjects; red symbols are context-dependent decay model; black and red lines are half Gaussians fit to experimental and model data, respectively). The p value is from a single-factor ANOVA (F[4, 55] = 18.21). Δθ° is probe orientation relative to exposure. (D) Decay of adaptation measured during reexposure blocks, plotted as in (C). Reexposure PD (mean over first eight trials immediately after probe) is measured in the original exposure orientation (R180°; see blue panels in A and B). Larger values indicate greater amounts of decay have occurred in the preceding probe block (Δθ°). Orange trace shows uniform decay predicted by the context-independent decay model. The p value is from a single-factor ANOVA (F[4, 55] = 9.83). Preprobe PD (mean over last eight trials immediately before probe; data not shown) did not vary significantly with probe Δθ° (ANOVA F[4, 55] = 0.01, p > 0.9). See Figure S2 for equivalent analysis of G0°.

To examine the potential effect of context on the decay of motor memory, we used error-clamp trials to prevent displacement of the handle, thereby clamping the kinematic error (*e*_n_) to zero (Figure 1C). This eliminates error-dependent memory formation (second term in Equation 1), allowing us to isolate memory decay (first term in Equation 1). Blocks of error-clamp trials were presented across a range of contexts (object orientations), and motor memory was examined before and after each block (Figure 2A).

Two groups of subjects performed an initial exposure block at a single exposure orientation: 180° (G180°, exposure at 180°; see Figure 2A) and 0° (G0°, Figure S2A). Consistent with our previous studies [7, 24], subjects rapidly adapted to the dynamics of the object during the initial exposure block, as shown by a progressive decrease in PD across trials (yellow panel, Figures 2B and S2B). Subjects then performed a series of probe blocks, each consisting of 20 error-clamp trials presented at a particular object orientation (green panel in Figures 2A and S2A). After each probe block, subjects were retested in the original exposure orientation for 18 (reexposure) trials to examine the decay of memory as a function of context (blue panel in Figures 2A and S2A).

During error-clamp probe trials, the compensatory force produced by subjects can be measured. The level of adaptation was quantified as the peak compensatory force divided by the force that would fully compensate for the object dynamics (Figures 2B and S2B). Consistent with our previous studies [7, 24], adaptation measured during probe blocks varied systematically across orientations and was highest at the exposure orientation (Δθ = 0° in Figures 2C and S2C), decreasing progressively as the relative probe orientation (Δθ) increased.

PD immediately before each probe block did not vary significantly with probe orientation, showing that subjects were in a comparable state of adaptation before each probe block (details of statistical tests are reported in the figure legends). PD on reexposure trials, immediately after each probe, provides our test for context-dependent memory decay. Current models predict that reexposure PD should not vary as a function of probe orientation. However, there was a highly significant effect of probe orientation on reexposure PD. Specifically, PD was greatest following error-clamp probe trials at the exposure orientation (Δθ = 0°, Figures 2D and S2D) and decreased progressively as the relative probe orientation (Δθ) increased. This indicates that memory decay was greatest during probe trials at the initial exposure orientation, with progressively less decay occurring at more distant probe orientations.

Because the above result cannot be reproduced by existing models (orange trace in Figure 2D), we developed a new model that included context-dependent terms for both memory formation (**b**_n_) and decay (**a**_n_):

(Equation 2) $$z_{n+1}=a_{n}⊙z_{n}+b_{n}e_{n}.$$

In the new model, a context-dependent function associated with the retention factor (**a**_n_) allows memory decay to vary with context (the ⊙ operator denotes elementwise vector multiplication).

We tested three variants of the model (see Supplemental Experimental Procedures). In each model, the context-dependent learning rate (**b**_n_) was implemented by a scaled and offset Gaussian centered on the current context [7]. In the first model, memory decay was assumed to be context independent, consistent with previous studies (Equation 1). The second and third models were variants of the context-dependent decay model (Equation 2). In these models, the retention factor (**a**_n_) was also implemented by a scaled and offset Gaussian (as for **b**_n_). In the first context-dependent decay model, the generalization functions for memory formation (**b**_n_) and memory decay (**a**_n_) had the same widths (the same SD of the underlying Gaussians). In the second context-dependent decay model, the widths of memory formation and decay were independent.

**Figure 3:**
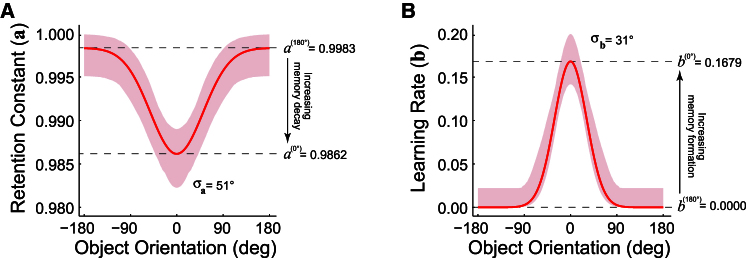
Gaussian Generalization Functions and Model Parameters for the Independent-Widths, Context-Dependent Decay Model (A) Model parameters for the retention factor (**a**_n_ in Equation 2) in the context-dependent decay model. The red trace shows the Gaussian generalization function obtained from fitting the mean subject data for both groups. The pink shading shows the 95% confidence limits obtained from a bootstrap analysis (see Supplemental Experimental Procedures for details). (B) Model parameters for the learning rate (**b**_n_ in Equation 2) in the context-dependent decay model, plotted as in (A). See also Tables S1 and S2 for additional modeling results and Figure S3 for control experiments.

The three models were fit to the experimental data, and the Bayesian information criterion (BIC) was used for model selection (see Supplemental Experimental Procedures). The BIC analysis strongly favored the model that allowed different widths for the generalization functions associated with memory decay and memory formation (see red and blue model fits in Figures 2 and S2). Examining the generalization functions in the model (Figure 3; see also Supplemental Experimental Procedures and Tables S1 and S2) shows that context-dependent memory decay had broader generalization (σ_**b**_ = 51°; Figure 3A) than context-dependent memory formation (σ_**a**_ = 31°; Figure 3B).

### Context-Dependent Decay for Novel Dynamic Force Fields

Numerous studies have examined motor memories using a task in which velocity-dependent force fields are applied to the hand [12–16, 26–29] (Figure 1D). The target-directed movements made by subjects are initially perturbed by the force field (Figure 1E). However, subjects adapt over the course of many trials with movements approaching their original unperturbed trajectories. We also examined context-dependent decay in this well-studied task, using our error-clamp paradigm (see Supplemental Experimental Procedures).

**Figure 4:**
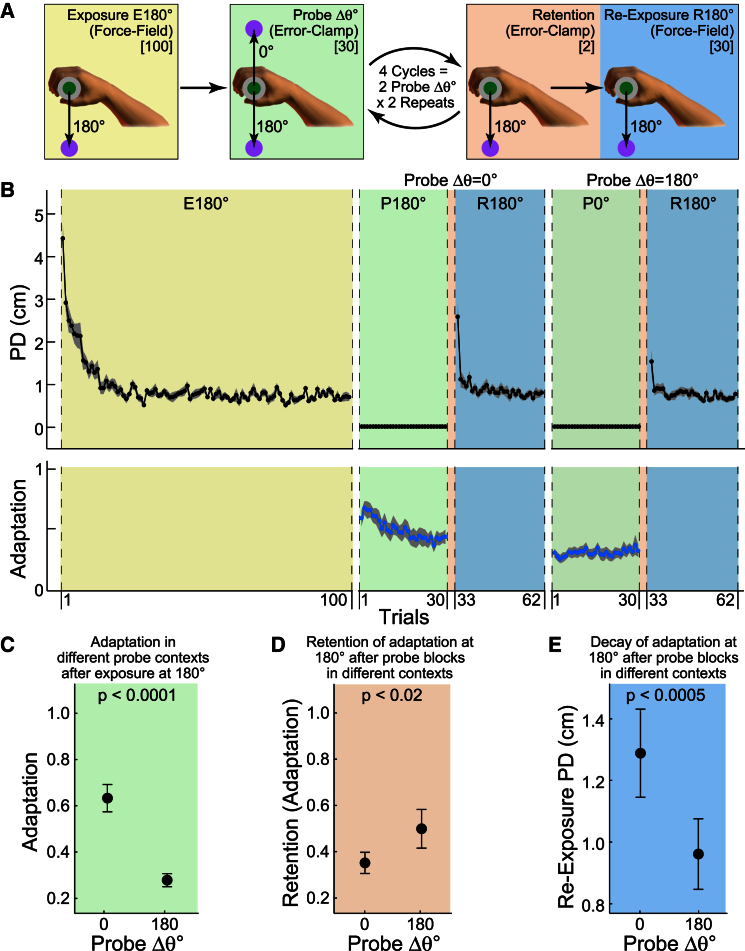
Context-Dependent Decay during Force-Field Adaptation for G180° (A) The experimental paradigm (trial counts in brackets). Subjects were initially exposed to the force field during reaching movements to the exposure target (180°) for 100 trials (E180°, yellow panel). They then performed four cycles of alternating probe and reexposure blocks. Probe blocks (green panel) consisted of 30 error-clamp trials presented at the original exposure target (Δθ = 0°) or a nonexposure target (Δθ = 180°). Reexposure blocks (blue panel) consisted of 30 force-field trials at the original exposure orientation (R180°). Two error-clamp trials at the exposure target were performed immediately before (data not shown) and immediately after (orange panel) each probe block. (B) Composite trial series for peak displacement (PD; upper panels, black trace) and adaptation (lower panels, blue trace) for G180° (mean across subjects; gray shading is SE). E, exposure (yellow panel); P, probe (green panels); R, reexposure (blue panels). Δθ° is probe target angle relative to exposure target. Orange panels show the sequence of two postprobe retention trials (data points are omitted for clarity). (C) Adaptation measured during probe blocks (green panels in A and B) after exposure at 180° (mean and SE across subjects). The p value is from a two-tailed paired t test (t[11] = 6.12). Δθ° is probe-target angle relative to exposure target. (D) Retention of adaptation measured during two error-clamp trials at the original exposure target immediately after probe blocks (see orange panels in A and B), plotted as in (C). Smaller values indicate greater amounts of decay have occurred in the preceding probe block (Δθ°).The p value is from a two-tailed paired t test (t[11] = 2.76). Preprobe adaptation (data not shown) did not differ significantly between probe targets (t test t[11] = 1.58, p > 0.1). (E) Decay of adaptation measured during reexposure blocks, plotted as in (C). Reexposure PD (mean over first eight trials immediately after probe) is measured at the original exposure target (see blue panels in A and B). Larger values indicate greater amounts of decay have occurred in the preceding probe block (Δθ°).The p value is from a two-tailed paired t test (t[11] = 4.91). Preprobe PD (mean over last eight trials immediately before probe; data not shown) did not differ significantly between probe targets (t test t[11] = 0.72, p > 0.4). See Figure S4 for equivalent analysis of G0°.

Two groups of subjects performed an initial exposure block reaching for a target in a single direction: 180° (G180°, Figure 4A) and 0° (G0°, Figure S4A). Adaptation to the force field was indicated by a progressive decrease in displacement of the hand (PD) from a straight line between the start position and the target (yellow panel, Figures 4B and S4B). Subjects then performed a series of probe blocks, which consisted of 30 error-clamp trials (green panel in Figure 4A). During error-clamp trials, the manipulandum simulated a mechanical channel that constrained hand movement to a straight line between the start position and the target (Figure 1F). As with the object manipulation task, error-clamp trials ensured that the kinematic error was zero and eliminated context-dependent memory formation. Probe blocks were presented at the 0° and 180° targets, thus representing the exposure (Δθ = 0°) and a nonexposure (Δθ = 180°) target. After each probe block, subjects were retested (reexposed) at the original exposure target to examine the decay of memory. In addition to error-clamp trials during probe blocks, subjects performed two error-clamp trials at the exposure target immediately before and after each probe block. This allowed us to measure the adaptation state of subjects.

We estimated the level of adaptation during probe blocks by dividing the peak force exerted by subjects on the channel wall [30] by the force which would have fully compensated for the force field. Consistent with previous studies, we found that adaptation was greater at the exposure target (probe Δθ = 0°) compared to the nonexposure target (probe Δθ = 180°) for both groups (G180°, Figure 4C; G0°, Figure S4C).

After subjects completed 30 error-clamp probe trials at either the exposure target or the nonexposure target, the retention of adaptation was measured using two error-clamp trials at the exposure target (orange panel in Figure 4A). Retention was significantly smaller (indicating greater decay) following probe trials at the exposure target (probe Δθ = 0°) compared to the nonexposure target (probe Δθ = 180°) for both groups (Figures 4D and S4D). In contrast, adaptation at the exposure target immediately before each probe block did not differ significantly between probe targets for either group.

The PD data confirms results obtained for adaptation described above. Specifically, PD immediately before each probe block did not vary significantly with probe target for either group, whereas reexposure PD immediately following each probe block did vary significantly for both groups (Figures 4E and S4E). These results indicate that context-dependent decay is also a feature of motor memories associated with learning novel force fields.

In both experiments, we used error-clamp trials to effectively remove kinematic errors. However, the error clamp was implemented with stiff springs, and very small kinematic errors remained. In one group of subjects in each experiment, probe context had a systematic effect on these small errors (see Supplemental Experimental Procedures). Previous studies have shown that small errors occurring during error-clamp trials do not drive deadaptation [30]. However, to verify that these small errors did not influence our findings and test their generality when subjects were exposed to the task dynamics at multiple contexts, we performed two control experiments. In the first control experiment, we manipulated the magnitude of the small errors associated with error-clamp trials by varying the spring stiffness. In the second control experiment, we exposed subjects to the dynamics at all probe orientations and varied the spring stiffness to equalize the small errors between probe contexts. In both cases, the context-dependent pattern of memory decay was still observed (see Supplemental Experimental Procedures and Figure S3).

## Discussion

We used a novel experimental approach to demonstrate that, contrary to previous assumptions [1–6, 8, 9], the decay of motor memory is highly context dependent. Specifically, the decay of memory associated with a given context is greatest for movements made in that context and decreases progressively for movements made in more distant contexts.

The finding that both memory formation and decay are greatest for the current context may seem paradoxical. However, we suggest that it provides a mechanism for optimizing performance. In motor tasks, memory decay is typically associated with reduced force output [10, 11]. Decay can thus be advantageous, because it tends to prevent the motor system from employing unnecessarily high force output. For example, when manipulating a given object, decay would prevent the application of unnecessarily high grip force [31]. However, decay will be disadvantageous if it results in performance errors. For example, an object will slip in the hand if grip force is reduced too much. These errors can be corrected by memory formation. For example, small slips (errors) during object manipulation result in an adaptive increase in grip force [32, 33]. Thus, simultaneous memory decay and formation, both of which are highest in the current context, allow the motor system to constantly probe whether its force output is unnecessarily high while still maintaining low error. An additional advantage of context-specific decay is that it preserves memories associated with distant contexts (for example, the required grip force for a different object). Indeed, it is difficult to regard memory decay at distant, nonactive contexts as anything but detrimental, especially because memory formation in these contexts cannot balance memory decay.

The context-specific effects we observe are graded such that similar contexts are also subject to the combination of memory formation and decay, whereas more distant contexts are unaffected. This enables the motor system to also optimize motor commands associated with contexts that are related. For example, when manipulating a particular object, it may be beneficial to optimize the motor memories associated with other similar objects (objects with similar mass or frictional properties). Our finding that the generalization function for memory decay is wider than for memory formation suggests that the balance between memory formation and decay may tend to favor decay for intermediate contexts. While more distant contexts are protected from both the formation and decay of memory, intermediate contexts may be subject to small amounts of decay. However, we note that although the difference in tuning width appears large (∼20°), simulations (not shown) suggest that the amount of decay occurring at intermediate contexts is small and rapidly corrected by memory formation when a particular intermediate context becomes active.

We have shown that context-dependent memory decay is present in two complementary tasks, one involving manipulating an object with familiar dynamics and the other involving reaching under novel dynamics. Showing context-dependent decay for both cases is important, as each task may engage different adaptation processes [7]. Specifically, during object manipulation, the structure of the dynamics is familiar and corresponds to commonly manipulated tools (such as hammers). Because subjects appear to have preexisting knowledge of such familiar dynamics [24], this is an example of parametric learning, in which only the mass of the object is unknown. In contrast, the force-field reaching task corresponds to an object with highly unusual dynamics. As such, adaptation requires learning both the structure (the equations relating motion to force) and the parameters (the values for the particular constants in those equations). The observation of context-dependent memory decay in both cases suggests that it may be a general mechanism in sensorimotor learning.

## Supplemental Information

Document S1. Figures S1–S4, Tables S1 and S2, and Supplemental Experimental Procedures

## References

[1] Thoroughman K.A., Shadmehr R. (2000). Learning of action through adaptive combination of motor primitives. Nature.

[2] Donchin O., Francis J.T., Shadmehr R. (2003). Quantifying generalization from trial-by-trial behavior of adaptive systems that learn with basis functions: theory and experiments in human motor control. J. Neurosci..

[3] Thoroughman K.A., Taylor J.A. (2005). Rapid reshaping of human motor generalization. J. Neurosci..

[4] Nozaki D., Scott S.H. (2009). Multi-compartment model can explain partial transfer of learning within the same limb between unimanual and bimanual reaching. Exp. Brain Res..

[5] Lee J.Y., Schweighofer N. (2009). Dual adaptation supports a parallel architecture of motor memory. J. Neurosci..

[6] Tanaka H., Sejnowski T.J., Krakauer J.W. (2009). Adaptation to visuomotor rotation through interaction between posterior parietal and motor cortical areas. J. Neurophysiol..

[7] Ingram J.N., Howard I.S., Flanagan J.R., Wolpert D.M. (2011). A single-rate context-dependent learning process underlies rapid adaptation to familiar object dynamics. PLoS Comput. Biol..

[8] Yokoi A., Hirashima M., Nozaki D. (2011). Gain field encoding of the kinematics of both arms in the internal model enables flexible bimanual action. J. Neurosci..

[9] Tanaka H., Krakauer J.W., Sejnowski T.J. (2012). Generalization and multirate models of motor adaptation. Neural Comput..

[10] Witney A.G., Goodbody S.J., Wolpert D.M. (2000). Learning and decay of prediction in object manipulation. J. Neurophysiol..

[11] Franklin D.W., Burdet E., Tee K.P., Osu R., Chew C.M., Milner T.E., Kawato M. (2008). CNS learns stable, accurate, and efficient movements using a simple algorithm. J. Neurosci..

[12] Shadmehr R., Mussa-Ivaldi F.A. (1994). Adaptive representation of dynamics during learning of a motor task. J. Neurosci..

[13] Gandolfo F., Mussa-Ivaldi F.A., Bizzi E. (1996). Motor learning by field approximation. Proc. Natl. Acad. Sci. USA.

[14] Shadmehr R., Brashers-Krug T. (1997). Functional stages in the formation of human long-term motor memory. J. Neurosci..

[15] Malfait N., Shiller D.M., Ostry D.J. (2002). Transfer of motor learning across arm configurations. J. Neurosci..

[16] Caithness G., Osu R., Bays P., Chase H., Klassen J., Kawato M., Wolpert D.M., Flanagan J.R. (2004). Failure to consolidate the consolidation theory of learning for sensorimotor adaptation tasks. J. Neurosci..

[17] Kagerer F.A., Contreras-Vidal J.L., Stelmach G.E. (1997). Adaptation to gradual as compared with sudden visuo-motor distortions. Exp. Brain Res..

[18] Ghahramani Z., Wolpert D.M. (1997). Modular decomposition in visuomotor learning. Nature.

[19] Krakauer J.W., Ghilardi M.F., Ghez C. (1999). Independent learning of internal models for kinematic and dynamic control of reaching. Nat. Neurosci..

[20] Miall R.C., Jenkinson N., Kulkarni K. (2004). Adaptation to rotated visual feedback: a re-examination of motor interference. Exp. Brain Res..

[21] Shadmehr R., Moussavi Z.M. (2000). Spatial generalization from learning dynamics of reaching movements. J. Neurosci..

[22] Mah C.D., Mussa-Ivaldi F.A. (2003). Generalization of object manipulation skills learned without limb motion. J. Neurosci..

[23] Mattar A.A., Ostry D.J. (2007). Modifiability of generalization in dynamics learning. J. Neurophysiol..

[24] Ingram J.N., Howard I.S., Flanagan J.R., Wolpert D.M. (2010). Multiple grasp-specific representations of tool dynamics mediate skillful manipulation. Curr. Biol..

[25] Howard I.S., Ingram J.N., Wolpert D.M. (2009). A modular planar robotic manipulandum with end-point torque control. J. Neurosci. Methods.

[26] Howard I.S., Ingram J.N., Wolpert D.M. (2008). Composition and decomposition in bimanual dynamic learning. J. Neurosci..

[27] Howard I.S., Ingram J.N., Wolpert D.M. (2010). Context-dependent partitioning of motor learning in bimanual movements. J. Neurophysiol..

[28] Tcheang L., Bays P.M., Ingram J.N., Wolpert D.M. (2007). Simultaneous bimanual dynamics are learned without interference. Exp. Brain Res..

[29] Tong C., Wolpert D.M., Flanagan J.R. (2002). Kinematics and dynamics are not represented independently in motor working memory: evidence from an interference study. J. Neurosci..

[30] Scheidt R.A., Reinkensmeyer D.J., Conditt M.A., Rymer W.Z., Mussa-Ivaldi F.A. (2000). Persistence of motor adaptation during constrained, multi-joint, arm movements. J. Neurophysiol..

[31] Westling G., Johansson R.S. (1984). Factors influencing the force control during precision grip. Exp. Brain Res..

[32] Johansson R.S., Westling G. (1984). Roles of glabrous skin receptors and sensorimotor memory in automatic control of precision grip when lifting rougher or more slippery objects. Exp. Brain Res..

[33] Johansson R.S., Westling G. (1987). Signals in tactile afferents from the fingers eliciting adaptive motor responses during precision grip. Exp. Brain Res..

