# Supplementary material for: Context-Dependent Decay of Motor Memories during Skill Acquisition
Source: Curr Biol. 2013 Jun 17;23(12):1107–12. doi: 10.1016/j.cub.2013.04.079 (PMC3688072; doi:10.1016/j.cub.2013.04.079)
Supplement: Document S1. Figures S1–S4, Tables S1 and S2, and Supplemental Experimental Procedures [file mmc1.pdf]

Current Biology, Volume *23*

Supplemental Information

Context-Dependent Decay of Motor Memories during Skill Acquisition

James N. Ingram, J. Randall Flanagan, and Daniel M. Wolpert

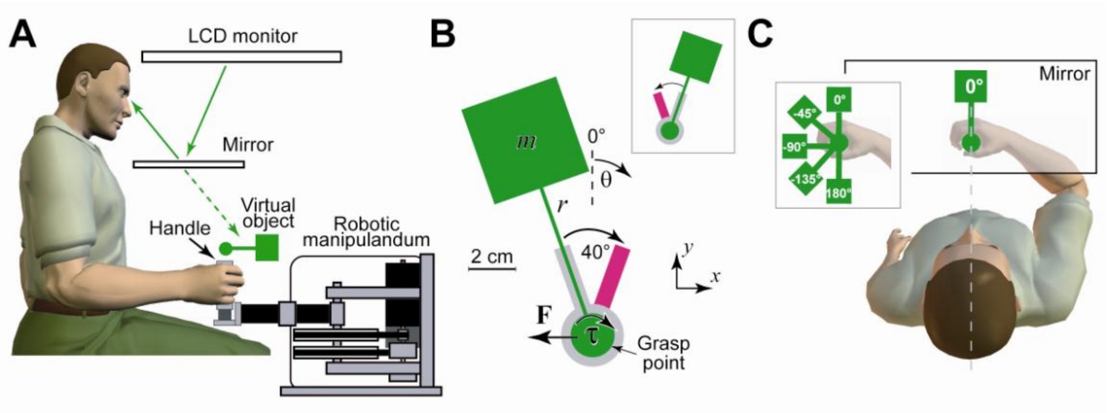

Figure S1, Related to Figure 1. The Virtual-Object-Manipulation Task

(A) Side view of the virtual reality system. Subjects are seated and grasp the handle of the WristBOT robotic manipulandum. An LCD monitor and mirror presents a virtual image of the object (green) and the task in the plane of movement. The image of the object tracks the translation and rotation of the handle in real-time and the WristBOT generates the forces and torques associated with the simulated object dynamics.

(B) The virtual object and visual feedback of the task. The task involves rotating the object (green) 40° from the starting angle (light grey bar) to a target angle (purple bar) while maintaining the circular handle of the object (the grasp point) within the home region (light grey disc). A clockwise trial is shown (the subsequent counter-clockwise trial is shown in the inset). The object is simulated as a mass (m) on the end of the mass-less rigid rod (r). Rotation (θ) generates a torque (τ) associated with the moment of inertia of the object. Rotation also generates a force ($\mathbf{F}$) associated with the circular motion of the mass.

(C) Top view showing visual feedback of the virtual object (green), which was projected over the subject's hand in the plane of movement. Visual feedback was consistent with grasping the object at its circular base. Dotted line shows subject's mid-sagittal plane which was aligned with the hand and the vertical rotation axis of the object. Inset shows top view of subject's hand overlaid with five different orientations of the object.

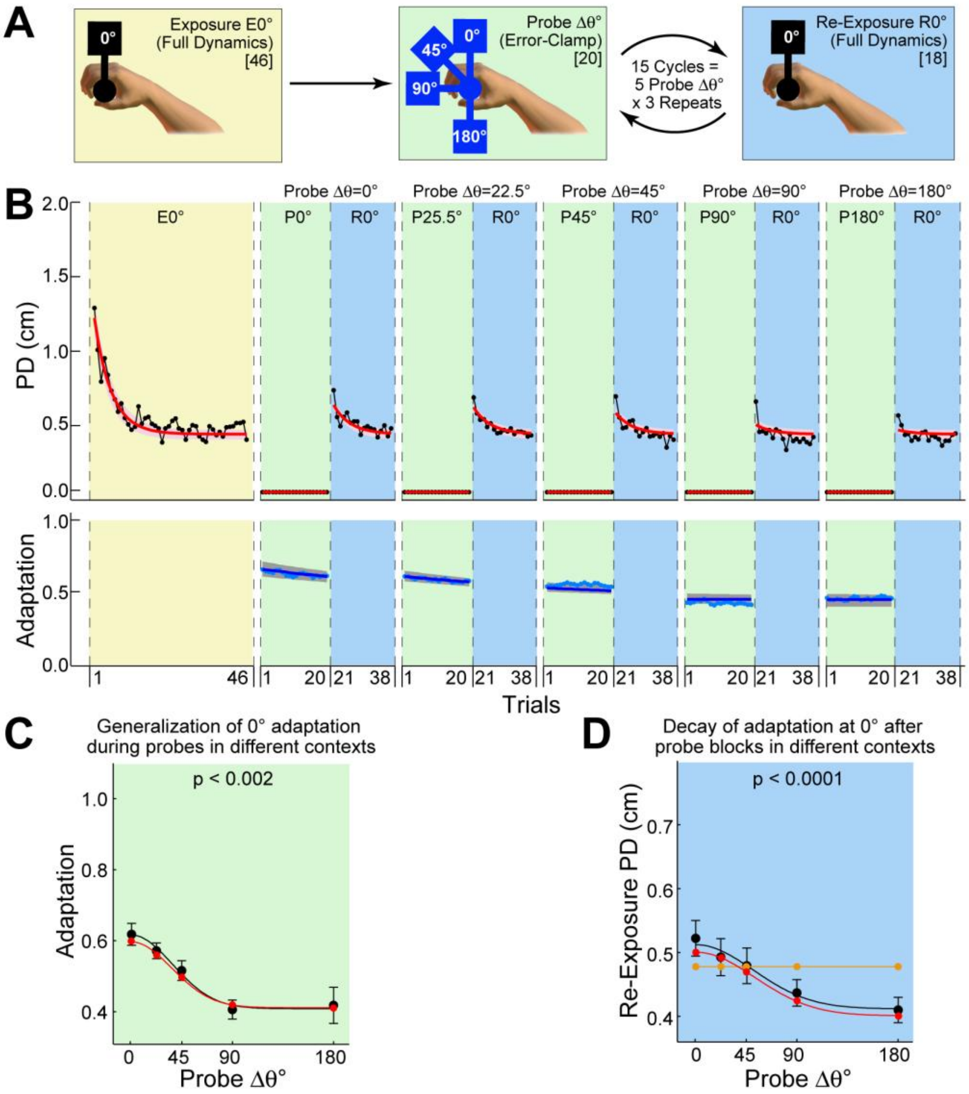

Figure S2, Related to Figure 2. Context-Dependent Decay during Object Manipulation for G0°

(A) The experimental paradigm (see main-text Figure 2 for details and an equivalent analysis of G180°).

(B) Composite trial series for peak displacement (PD; upper panels black trace) and adaptation (lower panels blue trace) for G0° (exposure at 0°) including the context-dependent decay model fit (red and dark blue lines; pink and grey shading shows 95% confidence limits for model fit). E=exposure (yellow panel), P=probe (green panels), R=re-exposure (blue panels). Δθ° is probe orientation relative to exposure.

(C) Generalization of adaptation measured during probe blocks (green panels in A and B) after exposure at 0° (black symbols are mean and SE across subjects; red symbols are context-dependent decay model; black and red lines are half Gaussians fit to experimental and model data, respectively). P-value for single-factor ANOVA ($F[4,55]=5.07$). Δθ° is probe orientation relative to exposure.

(D) Decay of adaptation measured during re-exposure blocks, plotted as in C. Re-exposure PD (mean over first 8 trials immediately after probe) is measured in the original exposure orientation (R180°; see blue panels in A and B). Larger values indicate greater amounts of decay have occurred in the preceding probe block (Δθ°). Orange trace shows uniform decay predicted by context-independent decay model. P-value for single-factor ANOVA

(F[4,55]=5.07). Pre-probe PD (mean over last 8 trials immediately before probe; data not shown) did not vary significantly with probe Δθ° (ANOVA $F[4,55]=0.25$, $p>0.9$).

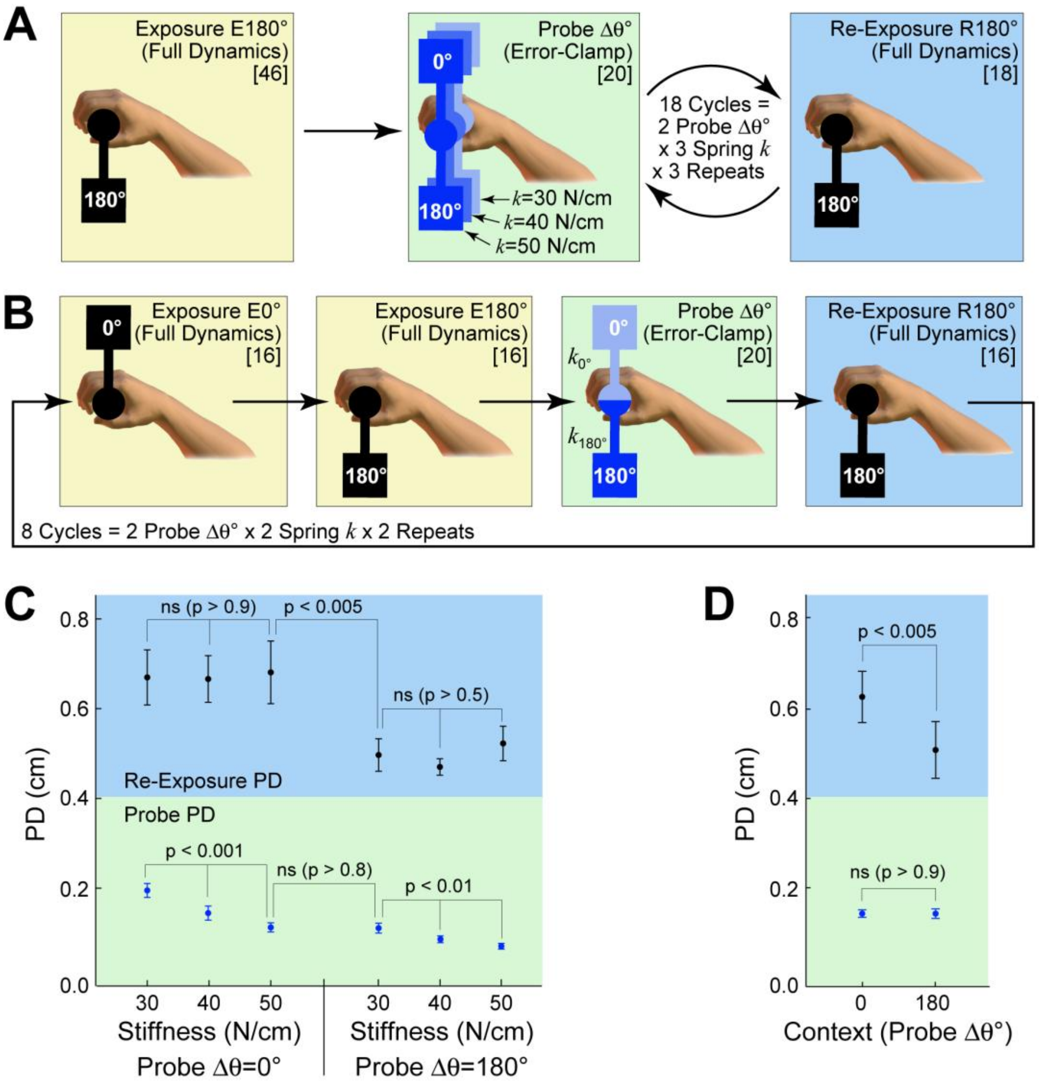

Figure S3, Related to Figure 3. Control Experiments

(A) Control Experiment 1 (see Supplemental Experimental Procedures for details). Subjects were initially exposed to the full object dynamics at the exposure orientation (E180° yellow panel). They then performed probe blocks consisting of 20 error-clamp trials (green panel) presented at 1 of 2 probe orientations (0° and 180°) and with 1 of 3 spring constants (k=30 N/cm, 40 N/cm, or 50 N/cm). After each probe block, subjects were re-exposed to the full object dynamics at the exposure orientation for 18 trials (blue panel).

(B) Control Experiment 2 (see Supplemental Experimental Procedures for details). Subjects were exposed to the full object dynamics at 0° and 180° (E0° and E180°, yellow panels). They then performed probe blocks consisting of 20 error-clamp trials also presented at 0° and 180° (green panel). Probe spring constants ($k_{0°}$ and $k_{180°}$) were adjusted so that error-clamp displacements were matched between the 2 probe orientations. After each probe block, subjects were re-exposed to the object dynamics at 180° for 16 trials (R180°, blue panel).

(C) Results for Control Experiment 1. Peak displacement (PD) during probe blocks (blue symbols on green panel) and subsequent re-exposure blocks (black symbols on blue panel) for the 2 probe orientations (Δθ°) and the 3 error-clamp stiffness levels. Symbols are means and SE across subjects. P-values are for statistical tests, as indicated (single-factor ANOVAs and paired two-tailed t-tests; see text for details; ns=non-significant).

(D) Results for Control Experiment 2. PD during probe blocks (blue symbols on green panel) and subsequent re-exposure blocks (black symbols on blue panel), plotted as in (C).

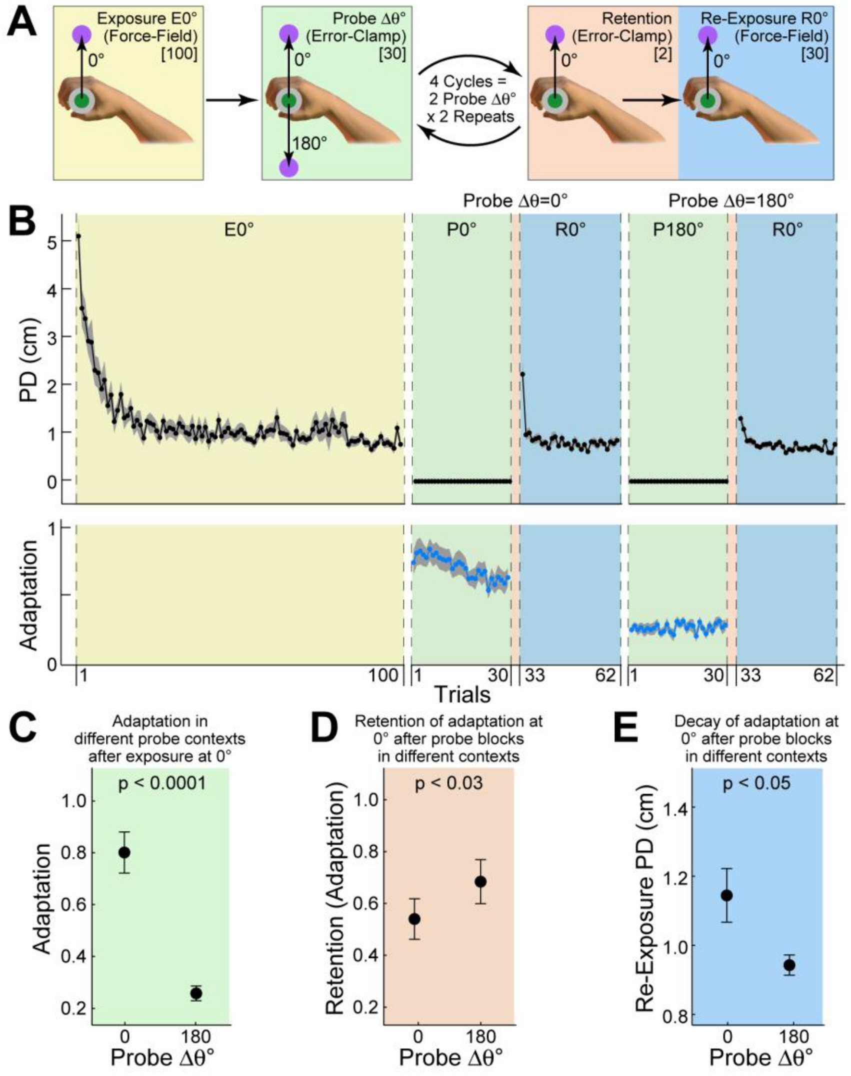

Figure S4, Related to Figure 4. Context-Dependent Decay during Force-Field Adaptation for G0°

(A) The experimental paradigm (see main-text Figure 4 for detail and equivalent analysis of G180°).

(B) Composite trial series for peak displacement (PD; upper panels black trace) and adaptation (lower panels blue trace) for G0° (exposure at 0°; means across subjects; grey shading is SE). E=exposure (yellow panel), P=probe (green panels), R=re-exposure (blue panels). Δθ° is probe target angle relative to exposure target. Orange panels show sequence of 2 post-probe retention trials (data points omitted for clarity).

(C) Adaptation measured during probe blocks (green panels in A and B) after exposure at 0° (mean and SE across subjects). P-value for two-tailed paired t-test ($t[11]=6.21$). Δθ° is probe target angle relative to exposure target.

(D) Retention of adaptation measured during 2 error-clamp trials at the original exposure target immediately after probe blocks (see orange panels in A and B), plotted as in C. Smaller values indicate greater amounts of decay have occurred in the preceding probe block (Δθ°). P-value for two-tailed paired t-test ($t[11]=2.56$). Pre-probe adaptation (data not shown) did not differ significantly between probe targets ($t[11]=1.58$, $p>0.1$).

(E) Decay of adaptation measured during re-exposure blocks, plotted as in C. Re-exposure PD (mean over first 8 trials immediately after probe) is measured at the original exposure target (see blue panels in A and B). Larger values indicate greater amounts of decay have

occurred in the preceding probe block ($\Delta\theta°$). P-value for two-tailed paired t-test ($t[11]=2.23$). Pre-probe PD (mean over last 8 trials immediately before probe; data not shown) did not differ significantly between probe targets ($t[11]=0.41$, $p>0.6$).

Table S1, Related to Figure 3. Model Parameter Values and 95% Confidence Limits

Parameter	Value	95% CL
$a^{0°}$	0.9862	0.9823 - 0.9890
$a^{180°}$	0.9983	0.9951 - 0.9999
$\sigma_{\mathbf{a}}$	51.4°	36.7 - 67.3°
$b^{0°}$	0.1679	0.1416 - 0.1995
$b^{180°}$	0.0000	0.0000 - 0.0221
$\sigma_{\mathbf{b}}$	31.2°	26.6 - 44.8°

Parameters values are for fitting the independent-widths context-dependent decay model (Model 3) to the mean trial series data across both groups of subjects (G0° and G180°) in Experiment 1 (see main-text Figure 2 and supplemental Figure S2). Confidence limits (CL) were calculated using a sub-sampling bootstrap (see Supplemental Experimental Procedures for details).

Table S2, Related To Figure 3. The R^2 Values for Model Fits

Model	Description	R^2	Sub-sample R^2	BIC%
1	Context-independent decay model	0.8956	0.8726±0.0101	0.0%
2	Context-dependent decay model with common tuning widths	0.9074	0.8840±0.0095	10.7%
3	Context-dependent decay model with independent tuning widths	0.9104	0.8868±0.0091	89.3%

The models were fit to the mean trial series data across both groups of subjects (G0° and G180°) in Experiment 1 (see main-text Figure 2 and supplemental Figure S2). The mean sub-sample R^2 values (±SD) for model fits associated with the sub-sampling bootstrap are also provided. The BIC% values indicate the percentage of sub-samples for which each particular model was selected by the BIC analysis (see Supplemental Experimental Procedures for details).

Supplemental Experimental Procedures

Subjects

In total, 72 university students participated in the study after giving their informed consent. A local ethics committee approved the study and subjects were naïve to its purposes.

Experiment 1—Object Manipulation

The first experiment was designed to examine the context-dependent pattern of decay across multiple object orientations after exposure to the familiar dynamics of an object at a single orientation. The virtual object manipulation task has been previously described [7, 24]. Briefly, subjects grasped the handle of the WristBOT robotic manipulation with their right hand (Figure S1-A) [25]. The WristBOT simulated the dynamics (forces and torques) of a hammer-like object, which consisted of a mass on the end of a rigid rod (Figure S1-B). A virtual reality display system provided visual feedback associated with the object and the task. The task involved rotating the object 40° between two visually-presented targets, with clockwise (CW) and counter-clockwise (CCW) rotations alternating across consecutive trials (Figure S1-B). Subjects were asked to maintain the handle as still as possible within the central home region as they rotated the object.

The object consisted of a point mass (1% of the subject's body mass) at the end of an 8 cm rigid rod (Figure S1-B). The task required subjects to produce a torque to rotate the object as well as a compensatory force to stabilise the handle. The compensatory force opposes the force associated with the circular motion of the mass (**F** in Figure S1-B; see also main-text Figure 1A), which would otherwise displace the handle. Full details of the object dynamics and the required compensation have been previously published [7, 24]. The visually displayed object (Figure S1-B) consisted of a circular handle (radius 0.5 cm) attached to a 4 cm square mass by an 8 cm rod (width 0.2 cm). The position and orientation of the object was determined by the position and orientation the WristBOT handle. The home region was a 1 cm radius disc and the start and end targets for rotation were oriented rectangles (0.6 by 2.5 cm) continuous with the home region (Figure S1-B).

Subjects performed blocks of trials consisting of CW and CCW rotations of the object between two targets. The angular midpoint between the targets defined the orientation of the object for that block of trials. The object could be presented and different orientations in each block (Figure S1-C). A trial began with the handle stationary within the home region and the rod of the object aligned with the start target. The movement was cued by a tone and the appearance of the second target. The trial ended when the subject had rotated the object to reach the second target. Subjects were required to make the movement within 400 ms. They were warned if they took longer and had to repeat the trial if the movement exceeded 500 ms. Rest breaks (45 s) were given every 3-5 minutes in all experiments.

Subjects experienced the torque associated with rotating the object on all trials. However, the forces generated by the manipulandum could vary according to three different trial types. On exposure trials, subjects experienced the forces associated with rotating the object (that is, they experienced the *full dynamics* of the object). Importantly, the forces generated on exposure trials cause the handle to displace (main-text Figure 1B), unless subjects generate a compensatory force to prevent displacement. On *error-clamp* trials (main-text Figure 1C), the manipulandum simulated a stiff two-dimensional spring centred on the handle position at the start of the trial (the spring constant was 40 N/cm unless otherwise stated). Error-clamp trials effectively eliminate kinematic errors [30] and prevent error-driven adaptation. Error-clamp trials also allow the compensatory forces generated by subjects to be

measured. Finally, on *zero-force* trials, the manipulandum did not produce any forces and the handle was free to move.

Subjects were randomly assigned to two groups (n=12 in each group) which were exposed to the dynamics of the object at either 0° (group G0°) or 180° (group G180°). Before the main experiment, subjects completed a familiarisation phase which consisted of 40 zero-force trials presented in blocks of 4 trials for each of 5 orientations (2 repeat blocks for each orientation). The 5 orientations presented during the familiarisation phase were the same as those presented during probe blocks for the main experiment (see below).

In the main experiment (Figure S2-A and main-text Figure 2A), subjects were first exposed to the object at 0° (group G0°) or 180° (group G180°) for 46 trials. They were then presented with multiple probe blocks which consisted of 20 error-clamp trials presented at 1 of 5 possible probe orientations. The 5 probe orientations were chosen for each group such that the relative probe orientations (Δθ relative to the exposure orientation) were the same for each group (probe Δθ of 0°, 22.5°, 45°, 90° and 180°). As such, for the 0° exposure group (G0°), probe orientations were 0°, -22.5°, -45°, -90° and 180° (Figure S2-A) and for the 180° exposure group (G180°), probe orientations were 180°, -157.5°, -135°, -90° and 0° (main-text Figure 2A). After each probe block, subjects were re-exposed to the full dynamics of the object for 18 trials at the original exposure orientation. Importantly, because re-exposure trials are presented at the original exposure orientation, they allow us to quantify the decay of memory which has occurred during the preceding probes at each orientation. As such, each block of 18 re-exposure trials is characterised by the relative orientation of the probe (Δθ) which immediately precedes it.

Probe orientations were presented in a pseudo-random order and each probe orientation was presented exactly once within a sequence of 5 probe blocks. Subjects performed 3 sequences of the 5 probe orientations (that is, 3 repeats per probe orientation). The experiment thus consisted of 15 cycles (5 probe orientations x 3 repeats per orientation = 15 cycles; Figure S2-A and main-text Figure 2A). Composite trial series for each subject were constructed by averaging trial data (see below) across the 3 repeats for each probe orientation and sorting probe blocks in order of increasing relative probe orientation (Δθ). Composite trial series consisted of the 46 initial exposure trials (yellow panels in Figure S2-B and main-text figure 2B) followed by 38 trials for each probe block. These 38 trials consisted of the 20 error-clamp trials at a given probe orientation (green panels in Figure S2-B and main-text figure 2B) followed by the 18 re-exposure trials (blue panels in Figure S2-B and main-text figure 2B). Composite trial series thus consisted of 236 trials (46 + 5 x (20+18) = 236).

Data Collection and Analysis

The position and orientation of the handle and the forces and torques generated by the manipulandum were recorded at 1000 Hz for offline analysis using Matlab (R14, The MathWorks Inc., Natick, MA, USA). Two measures characterized the trial-by-trial performance of the subjects during the task. On exposure trials, the peak displacement (PD) of the handle was measured, relative to its position at the start of the trial (main-text Figure 1B). On error-clamp trials, displacement of the handle was minimised (main-text Figure 1C), and instead the compensatory forces produced by subjects were measured. To quantify adaptation, we divided the peak force produced by the subject on error-clamp trials by the force which would have perfectly compensated for the perturbing dynamics of the object. As such, this measure has a value of 1 if subjects produce forces which exactly compensate for the dynamics of the object.

Composite trial series of PD and adaptation trial data were constructed for each subject as described above. Derived measures were also calculated from the PD and adaptation data associated with each probe orientation. Specifically, the

generalization of adaptation at each probe orientation was characterized by averaging adaptation data across the first 8 error-clamp trials for each probe (Figure S2-C and main-text Figure 2C). The decay of adaptation associated with each probe orientation was characterized by averaging PD data across the first 8 re-exposure trials which immediately followed each probe (Figure S2-D and main-text Figure 2D).

All statistical tests were performed using Matlab. All t-tests were paired and two-tailed and all ANOVAs were single factor.

The Models

We fit various state-space adaptation models to our data. The adaptation state is represented by a vector $\mathbf{z}$ which has elements corresponding to the different contexts (object orientations). The different possible contexts are represented by a context vector, $\boldsymbol{\theta}$, which has elements that correspond to the different visual orientations of the object. On the nth trial, the context is given by the index c_n such that the object orientation is $\boldsymbol{\theta}^{(c_n)}$ and the adaptation state associated with this context is $\mathbf{z}^{(c_n)}$. We use the superscript (i) to refer to the ith element of a vector.

For our experiment, the state ($\mathbf{z}$) and context vector ($\boldsymbol{\theta}$) have 16 elements covering all possible object orientations in 22.5° steps, such that $\boldsymbol{\theta}^{(i)} = 22.5i$, and c_n is the index (i) in these vectors (between 1 and 16) which corresponds to the current context. The state vector updates from one trial (n) to the next (n+1) according to:

$$\mathbf{z}_{n+1} = \mathbf{a}_n \odot \mathbf{z}_n + \mathbf{b}_n e_n \tag{S1}$$

where e_n is the scalar kinematic error, $\mathbf{b}_n$ is a vector mediating memory formation, $\mathbf{a}_n$ is a vector mediating memory retention (and hence decay) and $\odot$ denotes element-wise multiplication of the vectors. Importantly, the vectors $\mathbf{a}_n$ and $\mathbf{b}_n$ can vary from trial to trial depending on the current context c_n.

The kinematic error e_n (the peak displacement or PD of the handle), is calculated for exposure trials as follows:

$$e_n = \mathbf{d}^{(c_n)}(1 - \mathbf{z}_n^{(c_n)}) \tag{S2}$$

where $\mathbf{d}$ is vector of compliances for the different contexts. Values for $\mathbf{d}$ for different contexts (object orientations) have been determined in a previous study [7] and we used these values in the current study. Perfect compensation (zero error) for the current context, c_n, occurs when $\mathbf{z}_n^{(c_n)} = 1$. When $\mathbf{z}_n^{(c_n)}$ is less than 1, compensation is incomplete resulting in a non-zero error.

To simulate an experimental trial series, $\mathbf{z}$ was initialised to be the zero vector and only the time series of contexts $\{ c_1, c_2, c_3, \ldots \}$ and trial type is input to the model. Given the context and the current state of $\mathbf{z}$, the error can be simulated (Equation S2) and used to update $\mathbf{z}$ on exposure trials (Equation S1). On error-clamp trials, e_n is set to zero so that only the $\mathbf{a}_n$ vector affects the state update (Equation S1).

Model Variants

We consider 3 variants of the state-space adaptation model given by Equation S1. In all models, the learning-rate vector $\mathbf{b}_n$ is implemented by a generalization function which mediates context-dependent memory formation by weighting the influence of errors according to the context in which they occur. Specifically, the elements of $\mathbf{b}_n$ are set according to a scaled and offset Gaussian function centred on the current

orientation of the object (the current context). The form of the function is determined by 3 parameters w_1, w_2, and σ_b such that the ith element of $\mathbf{b}$ is:

$$\mathbf{b}_n^{(i)} = w_1 + w_2\mathcal{N}(\boldsymbol{\theta}^{(i)} - \boldsymbol{\theta}^{(c_n)}, \sigma_b) \quad \text{(S3)}$$

where $\mathcal{N}$ is the standard Normal probability density function:

$$\mathcal{N}(x,\sigma) = \frac{1}{\sqrt{2\pi}\sigma}e^{-\frac{x^2}{2\sigma^2}} \quad \text{(S4)}$$

which is applied to x (having taken the circular modulo). Therefore (for fits with w_2>0) the greatest update (learning) occurs for the current context with a decaying influence of errors at progressively more distant contexts. For consistency with previous studies, when reporting model parameters, we give $b^{(0°)}$ and $b^{(180°)}$, which are the values of $\mathbf{b}$ for the current context and the ±180° extremes, respectively.

The 3 model variants which were fit to the experimental data differed with regards to the form of the retention vector $\mathbf{a}_n$, as follows.

1. *Context-independent decay*. In this model, all elements of $\mathbf{a}_n$ are the same and independent of context:

$$\mathbf{a}_n = [a, a, \ldots, a]^T \quad \text{(S5)}$$

thus implementing uniform decay across all contexts. This is equivalent to previous models which have assumed context-independent decay (see main text for references). Model 1 has 4 parameters (3 associated with the learning-rate vector $\mathbf{b}_n$ in Equation S3 plus the scalar retention factor a in Equation S5).

2. *Context-dependent decay with common tuning widths*. In this model, $\mathbf{a}_n$ is context-dependent and (like $\mathbf{b}_n$) is implemented as a scaled and offset Gaussian:

$$\mathbf{a}_n^{(i)} = w_3 + w_4\mathcal{N}(\boldsymbol{\theta}^{(i)} - \boldsymbol{\theta}^{(c_n)}, \sigma_a) \quad \text{(S6)}$$

As with $\mathbf{b}_n$, when reporting model parameters for $\mathbf{a}_n$, we report $a^{(0°)}$ and $a^{(180°)}$. In this variant of the context-dependent decay model, σ_a and σ_b (in Equation S6 and S3, respectively) are constrained to be equal. Model 2 thus has 5 parameters. Note also that because w_4 can be negative, this model allows $a^{(0°)}$ to be less than $a^{(180°)}$. In this case, decay at the current context would be greater than the decay at more distant contexts.

3. *Context-dependent decay with independent tuning widths*. In this variant of the context-dependent decay model, the parameters specifying the standard deviations for the memory formation and retention generalization functions (σ_a and σ_b in Equation S6 and S3, respectively) could vary independently. Model 3 thus has 6 parameters.

Model Fitting

The three models were fit to the trial series data from Experiment 1. As with previous studies (Smith et al. 2006), the mean trial series across subjects was used for fitting because individual subject data is usually too noisy to obtain reliable fits. Specifically, we fit the trial series obtained experimentally for PD (measured on exposure trials) and adaptation (measured on error-clamp trials) to the simulated trial series for each model. For each probe context, we first averaged the data across the 3 repeats for each subject and then averaged these across subjects. The data were then used to

construct a single trial series from the smaller to largest probe orientation (Figure S2-B and main-text Figure 2B) and used to fit the model. This approach is justified because the PD immediately before probe blocks did not vary with probe orientation and therefore the order has only negligible affect on the fits.

Whereas kinematic error can be measured on exposure trials, on error-clamp trials the feedforward component of adaptation is measured. The adaptation state $\mathbf{z}$ in the model includes this feedforward component (feedforward compensatory forces) as well as components associated with limb impedance and feedback responses to kinematic error. We model the adaptation $\mathbf{z}$ as a linear function of the feedforward component ($\mathbf{z}_f$):

$$\mathbf{z} = k_1 + k_2 \mathbf{z}_f \tag{S7}$$

where k_2 is expected to be greater than 1. The parameters k_1 and k_2 were fit to the data.

Note that the measures on exposure trials (PD in cm) and error-clamp trials (adaptation is a dimensionless ratio) are in different units. To allow the model to be fit to the data in the same units (so as to ensure that trial type did not bias the fit) we performed all model fitting in error (PD) space. That is, on exposure trials, PD is measured experimentally and compared directly to the error (e) in the model. In contrast, for error-clamp trials from the model we use Equation S2 to determine the error (PD in cm) that would have occurred had the handle been free to move (that is, on a zero-force trial). For error-clamp trials from the experiment, we measured the feedforward adaptation ($\mathbf{z}_f$) and use Equation S7 convert $\mathbf{z}_f$, to $\mathbf{z}$ and Equation S2 to convert $\mathbf{z}$ to error (e). Therefore, we could generate a trial series of errors for both the experiment and the model and optimise the model parameters to minimize the mean squared difference between the two trial series.

The models were concurrently fit to the trial series for both groups of subjects (G0° and G180°). The parameters k_1 and k_2 (Equation S7) were fit separately for each group whereas all other parameters were common to both groups. Model parameters were estimated by a non-linear least-squares procedure performed in Matlab (`lsqnonlin`).

Confidence intervals for parameter estimates and the model trial series were calculated using a sub-sampling bootstrap (Politis et al. 1999). Specifically, the 12 subjects for each experiment were used to generate 495 unique sub-samples, each consisting of 8 subjects. The model was fit separately to the mean trial series data for each of the 495 unique sub-samples. The 95% confidence intervals were calculated as the 2.5 and 97.5 percentile values from the distributions for the parameters and the model trial series obtained across the 495 individual fits.

Model Selection

Model selection was performed using the Bayesian Information Criterion (BIC). The BIC for a particular model combines a “reward” for the goodness of fit with a “penalty” for the number of free parameters:

$$BIC = n \cdot \ln(\sigma_e^2) + k \cdot \ln(n) \tag{S8}$$

where σ^2_e is the variance in the residual errors of the fit, k is the number of free parameters and n is the number of data points (the number of trials). Taking the difference in BIC values for two competing models approximates half the log of the Bayes factor (Kass and Raftery 1995). A BIC difference of greater than 4.6 (a Bayes factor of greater than 10) is considered to provide strong evidence in favour of the model with the lower BIC value (Jeffreys 1998).

The BIC analysis was performed when fitting the models to the mean trial series data across all subjects and concurrently to both groups (G0° and G180°). The same BIC analysis was also applied separately to each sub-sample generated during the bootstrap (described above).

Supplemental Model Results

As reported in the main text, the BIC analysis (described above) selected the context-dependent decay model in which the widths for the generalization of memory decay and memory formation are independent (Model 3 as described above). Specifically, the difference in BIC values between the selected independent-widths model and the rejected single-width context-dependent decay (Model 2) and context-independent decay models (Model 1) were 9.6 and 60.1, respectively. This provides strong evidence in favour of the independent widths model (Jeffreys 1998).

The best-fit values for the parameters associated with memory formation ($b^{0°}$, $b^{180°}$, σ_b) and memory decay ($a^{0°}$, $a^{180°}$, σ_a) in the independent-widths model, along with the 95% confidence limits, are shown in Table S1 (see also main-text Figure 3). The R^2 values for fitting the 3 models to the mean trial series across both groups of subjects are shown in Table S2.

In addition to performing the BIC analysis on model fits to the mean trial series data, we also performed a separate BIC analysis for each bootstrap sub-sample (see Model Fitting in the Supplemental Experimental Procedures for full details). In the bootstrap analysis, the models were fit individually to 495 unique 8-subject sub-samples drawn from the 12 subjects (see Table S2 for the mean R^2 values for model fits across the 495 sub-samples). Specifically, the models were fit to the mean trial series for each sub-sample across both groups (G0° and G180°). The BIC analysis was applied separately to each bootstrap sub-sample in order to assess the robustness of the model selection result. The context-independent decay model was rejected by the BIC sub-sample analysis in all cases, and in 89.3% of sub-samples, the independent-widths model (Model 3) was selected (see Table S2). Moreover, when the tuning widths for memory decay (σ_a) and memory formation (σ_b) were compared on a sample-by-sample basis, the width for memory decay was wider than for memory formation in all cases, consistent with the results obtained for the entire subject pool.

Experiment 2—Force-Field Adaptation

The second experiment was designed to examine context-dependent decay during adaptation to a velocity-dependent curl-field. Subjects grasped the handle of a vBOT manipulandum [25] and made 12 cm point-to-point reaching movements between targets which were presented at either 0° or 180° (main-text Figure 1D). Whereas in the first experiment, the context was the orientation of the object, in the second experiment, the context was the movement direction (target angle). Subjects were randomly assigned to two groups (n=12 in each group) which were exposed to the force-field at the 0° target (group G0°; Figure S4-A) or the 180° target (group G180°; main-text Figure 4A). As such, subjects adapted to the force-field in one context (the exposure target) and decay was subsequently examined during movements made to the exposure target and the non-exposure target.

Subjects performed an initial 12 trials in the null field followed by 100 exposure trials during which the manipulandum generated a velocity-dependent curl-field (main-text Figure 1D). After each movement to the exposure target (0° for group G0° and 180° for group G180°), the vBOT passively returned the subject's hand to

the starting position ready for the next trial. During exposure trials the forcegenerated at the handle ($[F_x \quad F_y]$) depended on the velocity of the hand ($[\dot{x} \quad \dot{y}]$), as follows:

$$\begin{bmatrix} F_x \\ F_y \end{bmatrix} = k \begin{bmatrix} 0 & -1 \\ 1 & 0 \end{bmatrix} \begin{bmatrix} \dot{x} \\ \dot{y} \end{bmatrix} \tag{S9}$$

The direction of the field was determined by k which was -0.13 and +0.13 $\mathrm{N \cdot cm^{-1} \cdot s^{-1}}$ for clockwise (CW) and counter-clockwise (CCW) fields, respectively. Within each group the field direction was balanced so that half the subjects experienced a CW field and the other half experienced a CCW field.

As with Experiment 1, after the initial exposure to the task dynamics in a single context, subjects performed error-clamp probe blocks during which context-dependent decay was examined. Specifically, after the 100 initial exposure trials, subjects performed probe blocks each consisting of 30 error-clamp channel trials (main-text Figure 1F) at the 0° or 180° targets (Figure S4-A and main-text Figure 4A). On these trials the manipulandum simulated a mechanical channel (wall stiffness 40 N/cm) between the starting position and the target [30]. Each probe block was followed by 30 re-exposure trials at the original exposure target (0° for G0° or 180° for G180°). In addition, two error-clamp channel trials at the exposure target were performed immediately before and after each probe block. A total of 4 probe blocks were performed (2 repeats for each probe target), with the order randomised for each subject. The paradigm is illustrated for each group by the trial series in Figure S4-B (G0°) and main-text Figure 4B (G180°).

Performance on each trial was quantified by measuring the peak displacement (PD; main-text Figure 1E). PD was defined as the maximum perpendicular deviation of the hand from a straight line between the start position and the target. On error-clamp channel trials, the level of adaptation was quantified as the force exerted by subjects into the wall of the channel, at peak velocity, divided by the force which would fully compensate for the field. Composite trials series were constructed (as in Experiment 1) by averaging probe blocks across the multiple repeats for each probe target and then across subjects (Figure S4-B and main-text Figure 4B). As in Experiment 1, context-dependent decay was assessed by analysing PD during the re-exposure trials immediately following the probe (blue panel in Figure S4-A and main-text Figure 4A). Specifically, PD was averaged across the first 8 trials of each re-exposure block and across the 2 repeat blocks for each probe target. In addition, pre- and post-probe adaptation for each probe target was determined from the average of the 2 error-clamp channel trials at the exposure target immediately before and after each probe block. Specifically, the mean across pairs of pre- and post-probe error-clamp trials and then across the 2 repeat blocks for each probe target was calculated for each subject.

Control Experiments

In experiments 1 and 2, error-clamp trials were implemented by simulating a stiff spring with the robotic manipulandum, such that kinematic errors were minimised. Although the spring was very effective in minimising deviations of the handle (the mean PD across both groups during probe trials for Experiment 1 was 0.10±0.03 cm and for Experiment 2 was 0.20±0.04 cm), in one group of subjects for each experiment these small displacements varied systematically with probe context. Specifically, for group G180° in Experiment 1, probe PD varied with object orientation over a very small but significant range (PD range 0.03±0.01 cm, ANOVA $F[4,55]=0.68$, $p>0.6$ for G0°; PD range 0.05±0.02 cm, $F[4,55]=3.19$, $p<0.05$ for

G180°). Similarly, for group G0° in Experiment 2, probe PD varied with movement direction over a small but significant range (PD range 0.09±0.06 cm, paired t-test t[11]=5.06, $p<0.0004$ for G0°; PD range 0.03±0.03 cm, paired t-test t[11]=0.72, $p>0.4$ for G180°). To determine whether these small errors influenced our results, we performed two control experiments. In both experiments, we varied the stiffness of the error-clamp spring in order to manipulate the magnitude of the small errors associated with probe blocks.

Control Experiment 1—Variable Error-Clamp

Methods

The first control experiment was similar to group G180° from Experiment 1, except that probe trials were presented at only 2 orientations (0° and 180°) and the error-clamp spring constant was varied across different probe blocks (30, 40 and 50 N/cm; Figure S3-A). As in Experiment 1, subjects (an additional n=12) first completed 46 exposure trials at 180° followed by a number of cycles of probe and re-exposure blocks (Figure S3-A). In this case, subjects completed 18 cycles of probe blocks (2 probe orientations x 3 spring constants x 3 repeats = 18 cycles). The results were analysed as for Experiment 1.

Results

Results for Control Experiment 1 are summarised in Figure S3-C. As expected, varying the error-clamp spring stiffness had a systematic effect on the small displacements which occurred during probe blocks at both the exposure (180°) and the non-exposure (0°) orientations. Specifically, at the exposure orientation, probe PD varied significantly with spring stiffness (PD range 0.08±0.02 cm; ANOVA $F[2,33]=8.90$, $p<0.001$; blue symbols on green background for $\Delta\theta=0°$ in Figure S3-C). Similarly, at the non-exposure orientation, probe PD also varied significantly with spring stiffness (PD range 0.04±0.02 cm; ANOVA $F[2,33]=5.74$, $p<0.01$; blue symbols on green background for $\Delta\theta=180°$ in Figure S3-C). However, despite these systematic differences in probe displacements, re-exposure PD immediately after the probe did not vary with spring stiffness for either the exposure (ANOVA $F[2,33]=0.01$, $p>0.9$; black symbols on blue background for $\Delta\theta=0°$ in Figure S3-C) or non-exposure probes (ANOVA $F[2,33]=0.69$, $p>0.5$; black symbols on blue background for $\Delta\theta=180°$ in Figure S3-C). Moreover, when the small displacements associated with probe blocks were matched between the exposure and non-exposure orientations (paired t-test $p>0.8$ on PD for 50 N/cm at $\Delta\theta$ =0° versus 30 N/cm at $\Delta\theta$ =180°, as indicated for blue symbols on green background in Figure S3-C), re-exposure PD was still significantly greater for $\Delta\theta=0°$ versus $\Delta\theta=180°$ probes (paired t-test t[11]=3.60, $p<0.005$, as indicated for black symbols on blue background in Figure S3-C). Results for Control Experiment 1 thus show that small systematic differences in probe PD comparable to those observed in Experiment 1 do not lead to systematic effects on re-exposure PD. Moreover, even when probe PD are matched between contexts, context-dependent decay is still observed.

Control Experiment 2—Dual-Context Exposure

Methods

The second control experiment was similar to the Control Experiment 1, except that subjects were exposed to the dynamics of the object at both probe orientations. This allowed us to test whether the context-dependent effects found in Experiment 1 were still observed when subjects were exposed to the dynamics of the object at more than one orientation. In addition, we also adjusted the spring stiffness on a subject-by-subject basis in order to closely match the kinematic errors (PD) between the two probe orientations. This provided a further control for the possible effects of small kinematics errors associated with probe trials.

Twelve additional subjects were exposed to the object at 2 orientations (180° and 0°) and probe blocks were also presented at these same orientations. The experiment consisted of an exposure phase followed by a probe phase. In the exposure phase, subjects were exposed to the full dynamics of the object at 0° and 180° in alternating blocks of 16 trials which were each repeated 4 times. After each pair of blocks at 0° and 180°, 4 error-clamp trials were presented randomly at one of the exposure orientations. These error-clamp trials allowed the peak forces associated with each orientation to be determined. At the end of the exposure phase, this force data was analysed so that the spring constants for the subsequent probe phase could be individually set for each subject. Specifically, the spring constants $k_{0°}$ and $k_{180°}$ (Figure S3-B) where calculated such that probe displacements were matched between the 2 probe orientations.

During the probe phase of the experiment, as in previous experiments, blocks of error-clamp trials were used to probe for context-dependent decay. However, in this case, each block of 20 error-clamp probe trials (green panel in Figure S3-B) was immediately preceded by 2 blocks of 16 exposure trials, first at 0° and then at 180° (yellow panels in Figure S3-B). The subsequent probe blocks were pseudo-randomly presented at either 0° or 180°. The re-exposure block which followed each probe block was always presented at 180° (blue panel in Figure S3-B). As such, context-dependent decay was always determined relative to 180°, as in Control Experiment 1. The results were analysed as for Experiment 1.

Results

Results for Control Experiment 2 are summarised in Figure S3-D. Adjusting the spring constants ($k_{0°}$ and $k_{180°}$ in Figure S3-B) for each subject, based on the forces measured at each orientation during the exposure phase, successfully matched probe displacement (paired t-test t[11]=0.03, p>0.9, as indicated for blue symbols on green background in Figure S3-D). However, despite experiencing the same kinematic errors during probe blocks at each orientation, and despite exposure to the dynamics of the object at both probe orientations, re-exposure PD was larger immediately following Δθ=0° probe blocks compared to Δθ=180° probe blocks (paired t-test t[11]=3.87, p<0.005, as indicated for black symbols on blue background in Figure S3-D). Thus, as with the first control experiment, context-dependent decay was still observed in Control Experiment 2.

The results of Control Experiment 2 are also important because they rule out an alternative account for what we have interpreted as context-dependent decay. In this alternative, during error-clamp probe trials (where the error is zero), subjects may attempt to return to the non-zero error which they had experienced during the exposure trials immediately preceding the probe. This may occur, for example, if subjects develop a forward model during exposure trials which predicts a non-zero error. Importantly, such a model would be most developed for contexts close to where the errors are experienced (that is, the exposure context). This could lead to our observed context-dependent effects, which would not be decay, but rather an active error-driven process that is most developed for the exposure context. In Control Experiment 2, subjects were exposed to the dynamics at both 0° and 180°, and probe blocks were also presented at these two orientations. As such, any process attempting to restore errors to pre-probe values would apply equally to both probe orientations (as subjects would have similar expectations for a non-zero error at both). As a result, when subjects are exposed to the dynamics at the probe orientations, context-dependent decay should be abolished. However, this was not the case. Context-dependent decay was still observed (Figure S3-D), thus ruling out this alternative hypothesis.

Supplemental References

Jeffreys, H. Theory of probability. Oxford: Oxford University Press, 1998.
Kass, R.E., and Raftery, A. Bayes factors. *J Am Stat Assoc* 90: 773-795, 1995.
Politis, D., Romano, J., and Wolf, M. Subsampling. New York, NY: Springer-Verlag, 1999.
Smith, M.A., Ghazizadeh, A., and Shadmehr, R. Interacting adaptive processes with different timescales underlie short-term motor learning. *PLoS Biol* 4: e179, 2006.
